# The motor inhibitory network in patients with asymmetrical Parkinson’s disease: An fMRI study

**DOI:** 10.1007/s11682-021-00587-5

**Published:** 2022-01-12

**Authors:** Francis R. Loayza, Ignacio Obeso, Rafael González Redondo, Federico Villagra, Elkin Luis, José A. Obeso, Marjan Jahanshahi, Maria A. Pastor

**Affiliations:** 1 Neuroimaging Laboratory, Neurosciences Department, Center for Applied Medical Research (CIMA), University of Navarra, 31008 Pamplona, Spain; 2 Neuroimaging and BioEngineering Laboratory, Faculty of Mechanical Engineering, Polytechnic University (ESPOL), Guayaquil, Ecuador; 3 HM-CINAC, HM Puerta del Sur, 28938 Móstoles, and CEU-San Pablo University, 28003 Madrid, Spain; 4 CIBERNED, Instituto Carlos III, Madrid, Spain; 5 Department of Neurology, Clínica Universidad de Navarra, Pamplona, Spain; 6 Cognitive-Motor Neuroscience Group, Department of Clinical and Movement Neurosciences, UCL Queen Square, Institute of Neurology & The National Hospital for Neurology and Neurosurgery, London, WC1N 3BG UK

**Keywords:** Parkinson’s disease, Subthalamic nucleus, Imaging, fMRI, Inhibition, Stop-signal reaction time task, Functional connectivity, Dopamine

## Abstract

Recent imaging studies with the stop-signal task in healthy individuals indicate that the subthalamic nucleus, the pre-supplementary motor area and the inferior frontal gyrus are key components of the right hemisphere “inhibitory network”. Limited information is available regarding neural substrates of inhibitory processing in patients with asymmetric Parkinson’s disease. The aim of the current fMRI study was to identify the neural changes underlying deficient inhibitory processing on the stop-signal task in patients with predominantly left-sided Parkinson’s disease. Fourteen patients and 23 healthy controls performed a stop-signal task with the left and right hands. Behaviorally, patients showed delayed response inhibition with either hand compared to controls. We found small imaging differences for the right hand, however for the more affected left hand when behavior was successfully inhibited we found reduced activation of the inferior frontal gyrus bilaterally and the insula. Using the stop-signal delay as regressor, contralateral underactivation in the right dorsolateral prefrontal cortex, inferior frontal and anterior putamen were found in patients. This finding indicates dysfunction of the right inhibitory network in left-sided Parkinson’s disease. Functional connectivity analysis of the left subthalamic nucleus showed a significant increase of connectivity with bilateral insula. In contrast, the right subthalamic nucleus showed increased connectivity with visuomotor and sensorimotor regions of the cerebellum. We conclude that altered inhibitory control in left-sided Parkinson’s disease is associated with reduced activation in regions dedicated to inhibition in healthy controls, which requires engagement of additional regions, not observed in controls, to successfully stop ongoing actions.

**Supplementary Information:**

The online version contains supplementary material available at 10.1007/s11682-021-00587-5.

## Introduction

Patients with Parkinson’s disease (PD) have delayed motor inhibition relative to age-matched controls on the standard [Gauggel et al., 2004] and the conditional [Obeso et al., 2011a] versions of the stop-signal reaction time task (SSRT). The SSRT is abnormally prolonged in PD and dopaminergic medication seems to improve SSRT in early stages of the disease [Manza et al., 2018] but has no significant effects in later disease stages [Obeso et al., 2011b]. A key finding relevant to the current study was the demonstration in healthy participants that successful stopping was associated with significant activation of a right-hemispheric network involving the inferior frontal cortex (IFC), subthalamic nucleus (STN) and pre-SMA [Aron and Poldrack, 2006].

In light of evidence for the over-activity of the STN and under-activation of the pre-SMA during movement as pathophysiological features of PD [Bergman et al., 1990; Jahanshahi et al., 1995], together with deficits in inhibitory control [Gauggel et al., 2004; Obeso et al., 2011a], the aim of the present study was to probe the neural substrates of inhibitory deficits in PD. As stated above, inhibition depends primarily on a right-hemispheric network in healthy participants [Aron et al., 2007; Garavan et al., 1999; Hampshire et al., 2010; Ray Li et al., 2008; Rubia et al., 2003], that when perturbed recruit alternative regions [Obeso et al., 2013; Zandbelt et al., 2013]. Patients with right frontal lobe lesions [Aron et al., 2003] or left inferior frontal gyrus [Swick et al., 2008] but also PD patients after right subthalamotomy [Obeso et al., 2014a] show impaired inhibitory control on the stop-signal task.

To investigate right-hemispheric specialization for motor inhibition in PD, we compared performance of the stop-signal task with the right and left hand of PD patients with left predominant motor signs (i.e. with altered right-hemisphere functioning). Our aim was to identify specific alterations in BOLD activation during motor inhibition in patients with predominant left-sided PD when performing the stop-signal task. Rigorous selection of PD patients with highly asymmetric left-sided disease will partly guarantee that the right hemisphere network implicated in inhibitory control to be underactive, which would provide some indirect evidence of the causal involvement of the proposed right-hemisphere network in response inhibition [Aron and Poldrack, 2006]. Our predictions were that relative to healthy controls, PD patients would exhibit altered activation and connectivity in the inhibitory network, with under-recruitment of key regions of the right hemisphere network, in particular the pre-supplementary motor area (pre-SMA), IFC and STN when patients stopped movements with the most affected left hand.

## Methods

### Participants

Fourteen (7 male) right-handed PD patients were recruited at the Department of Neurology, Clínica Universidad de Navarra, with a clinical diagnosis of idiopathic PD, with a mean age of 54.7y. (SD = 8.5y.). All patients met the UK Brain Bank diagnostic criteria for PD (Hughes et al., 1992) and exhibited adequate improvement with levodopa and other anti-parkinsonian drugs. The Hoehn & Yahr Scale was used to define PD stage of our sample [Hoehn & Yahr, 1967]. The Unified Parkinson’s Disease Rating Scale (UPDRS- section III) was completed as a measure of disease severity when patients were “on” and “off” their usual medication. All patients had predominantly left-sided PD with moderate motor severity, with UPDRS scores for the left hemibody being significantly higher/more severe than for the right hemibody both “on” and “off” medication (see Table 1). Patients were treated in combination with levodopa and dopamine agonists reported as levodopa equivalent dose. Patients performed this experiment while taking their usual medication.

**Table 1:** Demographic and clinical details of the samples. Data are means with standard deviations given in brackets

	PD	Controls	*p*
Age (years)	54.7 (8.5)	57.6 (8.6)	*p* = .80
Sex (male / female)	7 / 7	12 / 11	*p* = .10
MMSE	29.4 (2.2)	30.0 (0.7)	*p* = .72
Handedness	49.1 (3.0)	49.9 (0.2)	*p* = .81
Disease duration (years)	6.8 (3.5)	N/A	N/A
BIS-11	46.36(14.2)	34.92(9.0)	*p* = .005
H&Y	2.18(0.5)	N/A	N/A
UPDRS (OFF) left/right hemi-body	22.4 (3.3)/ 7.36 (3.5)	N/A	*p* = .003
UPDRS (ON) left/right hemi-body	8.4 (2.8) / 4.5 (2.7)	N/A	*p* > .01
L-Dopa dosage (mg/day)	636.0 (343.9)	N/A	N/A

*PD: Parkinson’s disease, MMSE: mini mental state examination, UPDRS: Unified Parkinson’s disease rating Scale; UPDRS: Unified Parkinson’s Disease Rating Scale; mg: milligrams. Non-parametric tests were used for group comparisons (Mann–Whitney tests) and within-subjects (Kolmogorov–Smirnov tests)*

Twenty-three (11 male) right-handed healthy controls with mean age of 57.6 y (SD = 8.6 y) took part in the study. The control group was recruited from patient’s spouses and local volunteers. None of the controls had any neurological disorder or history of psychiatric illness, drug or alcohol abuse and none were taking any medication as measured during the interview by a specialized neurologist. Information about the PD patients and the controls is presented in Table 1.

Patients and controls were screened for cognitive status with the Mini-Mental State Examination [MMSE; Folstein et al., 1975] performed by an specialized neuropsychologist. Screening for clinical depression on the Beck Depression Inventory [BDI; Beck et al., 1961] was done. The Barratt Impulsiveness Scale (BIS-III) was used to measure impulsivity [Patton et al., 1995]. Hence, the inclusion criteria consisted of preserved cognitive status, non-depressed, right-handed (measured by the Edinburgh Inventory [Oldfield, 1971] and (patient only) predominant left-sided motor signs. Exclusion criteria were depressed status and history of previous neurological or psychiatric diseases, and brain structural changes (head injury, MRI artifacts and cerebrovascular alterations visible at MRI scan). The study was approved by the University of Navarra Research Ethics Committee. All participants gave written informed consent prior to scanning.

### Stop-signal task

**Fig. 1:**
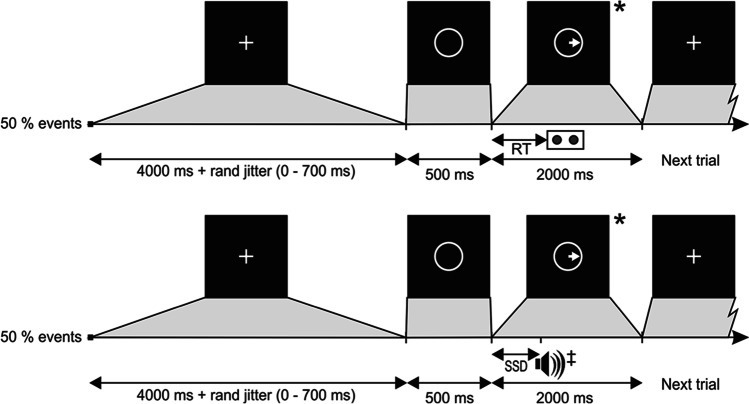
The stop signal task sequence of events of the two types of trials. 1) “Go” trials, where the participant is requested to press the left or right button on presentation of a left or right pointing arrow respectively presented in the (*) block. 2) “Stop” trials where the participant is requested to inhibit the movement when a beep (‡ stop signal) is presented after the Go signal. The stop signal delay, the interval between the Go and Stop signals, was varied between 50 and 450 ms and adjusted using a staircase tracking procedure depending on the reaction time and the participant’s success/failure in motor inhibition

The stop-signal task allows measurement of how quickly a participant can initiate a response (Go trials) and how well a participant can inhibit an already initiated response (Stop trials). Therefore, the stop-signal task consists of a random combination of Go and Stop trials. The participant was requested to look at a circular fixation point; a Go stimulus was presented in the middle of the screen 500 ms after presentation of a fixation circle. On Go trials, a left or right pointing arrow was presented during 2000 ms in the center of the screen and participants had to respond as fast as possible using their index or middle fingers to press a left or right key respectively. On Stop trials (40% of all trials), a stop-signal (50 ms duration beep) was presented after the left or right pointing arrow with a variable stop-signal delay (SSD). When a stop-signal was presented after the arrow (Go signal), the participants had to stop their response. The SSD values ranged from 50 to 450 ms and changed dynamically across trials contingent on the participant’s behavior. The staircase procedure operated with the SSD starting value selected randomly from one of the three time windows from the practice session (100–150-200–250), (150–200-250–300) or (200–250-300–350) ms. Successful inhibition of a response on a Stop trial required the next Stop trial inhibition presentation to be more difficult by increasing the stop-signal delay by 50 ms. By contrast, when the response was not successfully inhibited and a motor response was produced, on the next Stop trial, the SSD was decreased by 50 ms to facilitate successful inhibition. The algorithm performed the staircase procedure during the next 16 Stop trials and then repeated this procedure four times considering the non-used SSD starting values. The staircase tracking procedure ensured convergence to P (inhibit) around 50% by the end of the 16 blocks for each hand or session. This allowed us to obtain measures of each individual’s mean SSD when the probability of successfully inhibiting the motor response is at 50%. This is required to estimate the participants’ SSRT. For each session, left and right pointing arrows were presented pseudo-randomly and performance with the right or left hands was counterbalanced in each group. Figure 1 shows the sequence of events on Go and Stop trials.

Participants were instructed to focus on responding to the Go signal by pressing the correct response key (left or right) as fast and accurately as possible; while at the same time, they should also pay attention to the possible stop-signal (beep) on some trials and try to withhold their response on such trials. In addition, participants were informed that, due to the variable nature of the SSD, it would not always be possible to stop their response. Finally, participants were specifically instructed not to let their performance on the stopping task interfere with their performance on the Go task and, in particular, they were asked not to delay their performance on the Go task in order to improve their chances of stopping after presentation of a stop-signal.

### Design

A mixed design was used with group (LPD versus controls) as the between groups variable and performing hand (right versus left) as the within subject repeated measures factor.

### Procedure

All participants completed two separate fMRI counterbalanced sessions using either the right and left hand, responding using the two-button box. Clinical and behavioral assessments were conducted before the scanning during a full-day of testing. Before scanning session, participants had 20 practice trials per hand. This test session was used to obtain the first SSD convergence value, and then employed to calculate the time window composed by the four SSD starting values which was used during the fMRI. All patients were in the “on” state, and all the experiments were performed in afternoon sessions.

### Behavioral data analysis

To test for group and laterality effects on the stop-signal task performance, 2-way ANOVAs with Hand (right vs. left) and Group (patients vs. Controls) on the principal task measures were completed. Post-hoc independent t-tests [type I error-corrected, Benjamini & Hochberg, 1995] were used to compare the two groups on the measures derived from the stop-signal task. For variables showing non-normal distributions, non-parametric tests were used to compare performance across groups (i.e. % and error data; using IBM SPSS 21).

The SSRT was computed using the integration method [Verbruggen et al., 2019], as some participant’s inhibition probability was different from 50%. We first rank ordered the correct Go RTs. For each participant, the *n*th Go RT value was obtained by multiplying the total number of Go Trials by the probability of responding on Stop trials. Following previous works [Aron et al., 2007; Aron and Poldrack, 2006], the mean SSD was averaged from the mean values for the last six moves in each of the four staircases when the participant had converged on 50% inhibition and then subtracted from the *n*th Go RT value to compute the integration SSRT (iSSRT).

#### fMRI

Imaging was performed using a 3-Tesla scanner (Trio-TIM, Siemens AG, Erlangen, Germany) equipped with a 12-channel head coil array. Visual stimuli were projected onto a screen behind their head. Scripts for stimulus presentation and response recording were developed using Cogent (Cogent 2000, Wellcome Department of Imaging Neuroscience, UCL, London, UK) and Matlab v7.9 (Mathworks Inc., Natick, USA). Eye movements were monitored and registered using an eye-tracking device (ASL, Bedford, MA) for controlling sleepiness. A T2*-weighted (Echo Planar Imaging-EPI) was used to acquire ~ 300 volumes of the experiment. Each volume comprised 45 transverse slices with a 15% gap, resolution = 3 mm isotropic, echo time = 30 ms and repetition time = 3.0 s covering the entire brain. We also acquired a high resolution anatomical with an MPRAGE sequence and a 2D FLAIR image. Data were analyzed using “Statistical Parametric Mapping” software, version SPM12 (Wellcome Department of Imaging Neuroscience, UCL, London, UK). More details of the fMRI acquisition and data analysis are provided as supplementary material.

The scanning session consisted of 64 Stop, 64 Go and 32 Idle trials. Idle trials were presented randomly in each block; represent the baseline and consisted of a time period without activity of equal duration to Go events, containing components of movement preparation and attention while participants were waiting for either Go or Stop events. Trials were presented in 16 pseudo-randomized blocks containing 4 Go, 4 Stop and 2 idle events. The duration of Go events was 4000 + jitter (0–750) + RT ms (RT: response-time); for Stop-Inhibit events was 4000 + jitter (0–750) + 2000 ms and for Stop-Respond, Go and Idle events were 4000 + jitter (0–750) ms.

Despite the fact that head movements and imaging artifacts were controlled using the vacuum cushion available in the scanner, we added to the model the six movement regressors to minimize any effect of movement on the BOLD results. Thus, at the first level, the time series of each participant was modeled with the following tasks as an event related design: Go, Stop-Inhibit, SSD regressor (using the last 6 moves of the staircase, as in [Aron et al., 2007; Aron and Poldrack, 2006]), Stop-Respond, Idle, Errors and the six movement regressors; convolving each event with a canonical double gamma as a hemodynamic response function (HRF). Onset times for each event corresponded to the presentation of the visual cue (arrow), not discriminating between key presses. At this level, we estimated the following contrasts of interest for each participant and hand: Go vs. Idle and Stop-Inhibit vs. Idle, Stop-Inhibit vs. Go and the SSD regressor.

At the second level, for each contrast of interest we used two-way ANOVA with between-group factor (LPD and controls) and within-group factor (left or right hand). To minimize the variability between patients, we added the LEDD as a nuisance variable in the patients group. Additionally, to minimize the impulsivity variable between groups, the total BIS-11 impulsivity was added as a second nuisance factor at this level. Finally, only the contrasts for the SSD regressor were also modeled with the SSRT as a nuisance variable. We tested for each contrast between group differences in the BOLD signal. For the contrasts where the main effects were significant we created a post-hoc test to explain the main effects corrected using Bonferroni’s method. Additionally, to find whether patients present dysfunction in brain regions during the more difficult trials and equally evident for left and right hands, a conjunction analysis was performed. We also obtained the Percent Signal Change (PSC) to explain the contrast effects for both hands.

To test the functional connectivity of the STN, we performed a Psychophysiological Interaction analysis (PPI) [Friston et al., 1997] with the objective to assess variations in effective connectivity of the STN during the Stop-Inhibit vs. Idle events (psychological factor) [Friston, 1994][Ashburner & Friston, 2005]. We selected two seeds: one from the left and other from the right STN using pick-atlas [Maldjian et al., 2003]. To identify the brain regions which showed changes in functional connectivity from each seed and hand, we followed the procedure described by Gitelman et al., [Gitelman et al., 2003] using four independent matrices for each participant (left STN left hand, left STN right hand, right STN left hand and right STN right hand). Contrasts obtained in the individual PPI analyses for each matrix were introduced in a two-way ANOVA with factors group and hand. For all image results we used *p* < 0.005 FDR cluster corrected [Genovese et al., 2002]. Figures were performed using MricroGl (http://www.mccauslandcenter.sc.edu/mricrogl/) and Caret (http://brainvis.wustl.edu/wiki/index.php/Caret:About).

## Results

### Response inhibition did not differ between most affected and less affected side in LPD

**Fig. 2:**
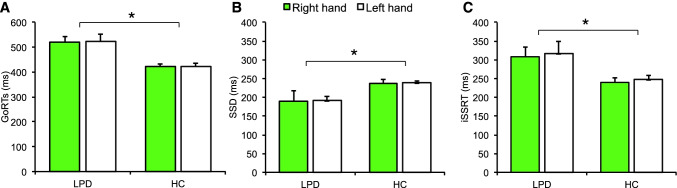
The mean Go RTs (**A**), stop signal delay (SSD; **B**) and stop-signal reaction time (**C**) derived using the integration stop-signal reaction time (iSSRT) for the patients with left predominance of arkinson’s disease (LPD) and healthy controls (HC) when completing the task with their right or left hands

Correct inhibition was achieved at 61% (LPD patients) and 68% (controls) probabilities without statistical differences between groups (Table 2; z = -1.33, *p* = 0.18) and both showing expected cumulative probabilities to fail stopping as SSD values increased (see Figure S2). The mean SSD comparison showed that LPD patients achieved successful inhibition with significantly lower (easier) SSD values than controls (Table 2 and Fig. 2) [*t*_(38)_ = 26.56, *p* < 0.001], independent of hand of performance.

**Table 2:** Means and standard deviation (in brackets) for the measures of interest on the stop signal reaction time task for patients with left-sided Parkinson’s Disease (PD) and for healthy controls performing the task with their right or left hands

Measure	PD		Controls		stats
	Right hand	Left hand	Right hand	Left hand	
Go RT (ms)	518.73 (95.4)	522.87 (109.9)	422.98 (52.7)	421.67 (65.2)	** + *p* < *.05*** *# p* > *.05* *¬ p* > *.05*
Stop Inhibit (%)	61.60 (16.7)	62.04 (16.9)	59.05 (13.5)	60.20 (12.1)	*¢ p* > *.05*
Stop Respond RT (ms)	422.82 (75.6)	435.49 (112.2)	381.67 (45.0)	373.01 (52.2)	+ *p* > *.05* ***# p* < *.05*** ***¬ p* < *.05***
Go errors	0.85 (1.2)	1.00 (1.5)	0.57 (1.2)	0.73 (0.9)	*¢ p* > *.05*
Stop Signal Delay (ms)	189.32 (19.3)	191.05 (40.1)	236.96 (48.3)	238.27 (27.5)	** + *p* < *.05*** *# p* > *.05* *¬ p* > *.05*
Stop Signal Reaction Time (ms)	307.34 (103.3)	316.70 (123.3)	239.86 (60.3)	247.37 (53.3)	** + *p* < *.05*** *# p* > *.05* *¬ p* > *.05*

*ms: milliseconds; mixed ANOVA results in statistics column show Group (* +*), Hand (#) or their interaction (¬) effects. Non-parametric tests were used in percentage and error comparisons (¢)*

A 2-way ANOVA with Hand (right vs. left) and Group (patients vs. Controls) on Go RTs showed a significant Group effect [*F*_(1,38)_ = 8.15, *p* = 0.007] indicating that patients were significantly slower than controls [*t*_(38)_ = 4.10, *p* < 0.001] independent of hand. No significant Hand x Group interaction was found [*F*_(1,38)_ = 0.60, *p* = 0.44] and the main effect of Hand was not significant either [*F*_(1,38)_ = 0.53, *p* = 0.46]. For Stop-Respond trials, a 2-way ANOVA with Hand (right vs. left) and Group (patients vs. Controls) on Stop-Respond RTs showed a significant Hand x Group interaction [*F*_(1,38)_ = 37.00, *p* = 0.01] and main effect of Hand [*F*_(1,38)_ = 5.55, *p* = 0.02] without a significant Group effect [*F*_(1,38)_ = 2.43, *p* = 0.12]. This was due to significantly longer Stop-Respond RTs for patients compared to controls with the left [*t*_(38)_ = 2.41, *p* = 0.02] and right hands [*t*_(38)_ = 2.16, *p* = 0.03], while faster compared to Go RT across groups and hands (Supplementary material). The differences between groups in errors was not significant [z = -1.84, *p* = 0.13]. Thus, the overall response speed was slower in the patient group but there were no significant group difference in accuracy.

A 2-way ANOVA with Hand (right vs. left) and Group (LPD vs. controls) on SSRT was performed. No significant Hand x Group interaction [*F*_(1,38)_ = 1.22, *p* = 0.27] or main effect of Hand [*F*_(1,38)_ = 0.66, *p* = 0.42] was found. As expected, there was a significant Group effect [*F*_(1,38)_ = 6.40, *p* = 0.016] with significantly longer iSSRTs in LPD patients, indicating worse/delayed motor inhibition than controls [*t*_(38)_ = 2.61, *p* = 0.01]. In summary, patients and controls showed significant differences on the Go RTs, SSD, iSSRT and Stop-Respond RTs but without significant main or interaction effects of Hand across most measures (Table 2 and Fig. 2).

### Imaging results: local and network effects of LPD

#### Stop-Inhibit trials

**Fig. 3:**
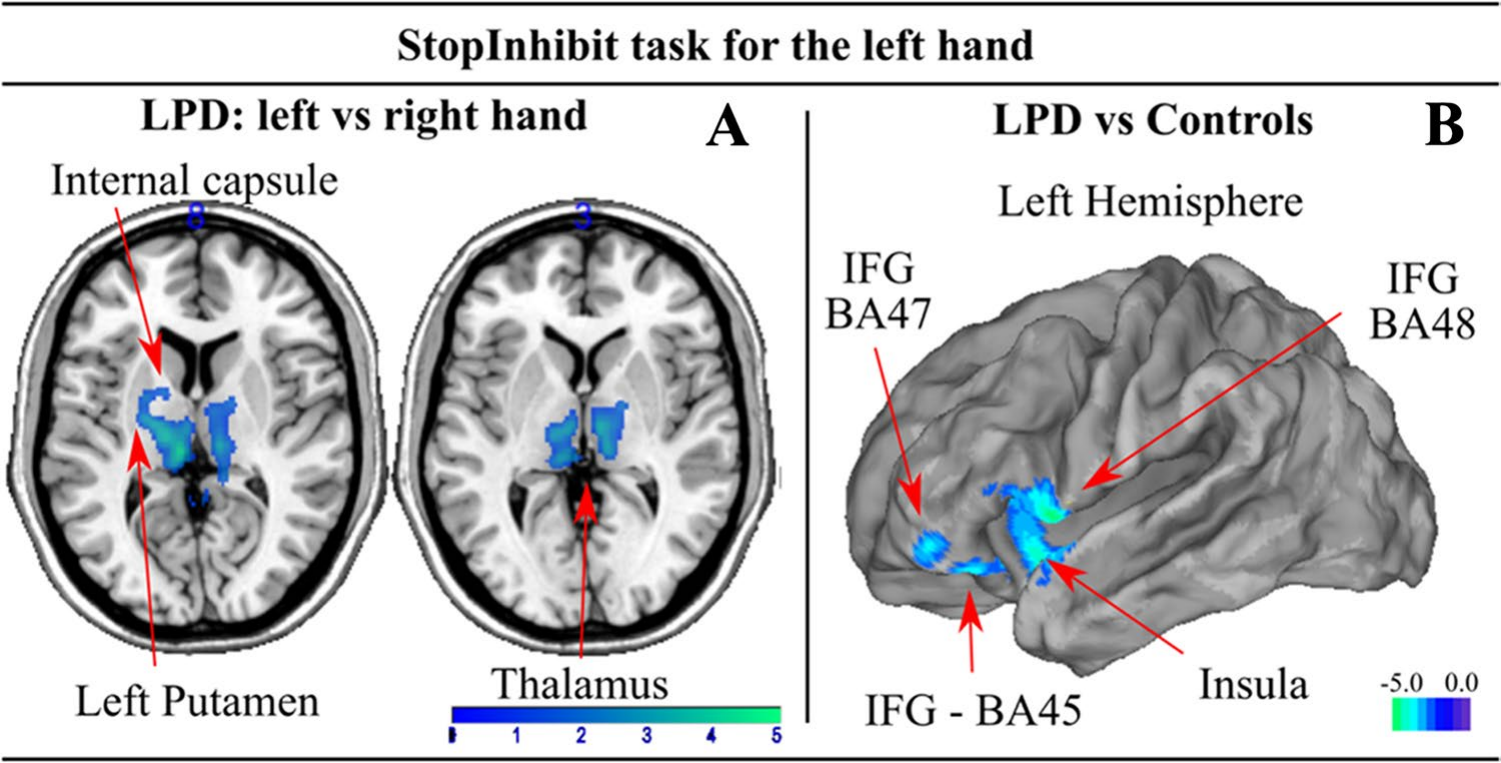
Brain regions under-activated in left-sided Parkinson’s disease patients compared to healthy controls during the successful inhibition condition for the most affected left hand. Panel **A** shows significant underactivations when comparing left vs. right hand in the patients group. We did not find significant differences between hands for the control group. Panel **B** depict brain regions underactivated when comparing the left hand with the control group during Stop-Inhibit task. We did not find significant differences for the right hand for this contrast. No significant hyperactive regions prevailed threshold

For the Stop-Inhibit trials (successful inhibition) we found significant main effects in our sample (Fig. 3B, Supplementary Table 1). Post-hoc comparisons showed differences in BOLD signal between patients and controls only for the most affected left hand, marked by significantly less activation relative to controls in the left IFG BA45-47 and 48 (pars triangularis and orbitalis) extending to temporal pole and anterior insula lobe.

In order to know whether dysfunction found in the LPD group is unique to a particular hand during the response inhibition, we used the successful inhibition contrast to test differences between hands. While no differences were found in the control group, the LPD patients exhibited a significant difference between hands showing underactivation along bilateral thalamus, extending to the putamen and pallidum during Stop-Inhibit trials (Fig. 3A, Supplementary Table 2). This result indicates a preferential underactivation of basal ganglia regions when using the most affected hand for motor inhibition.

#### Go vs. Idle trials

**Fig. 4:**
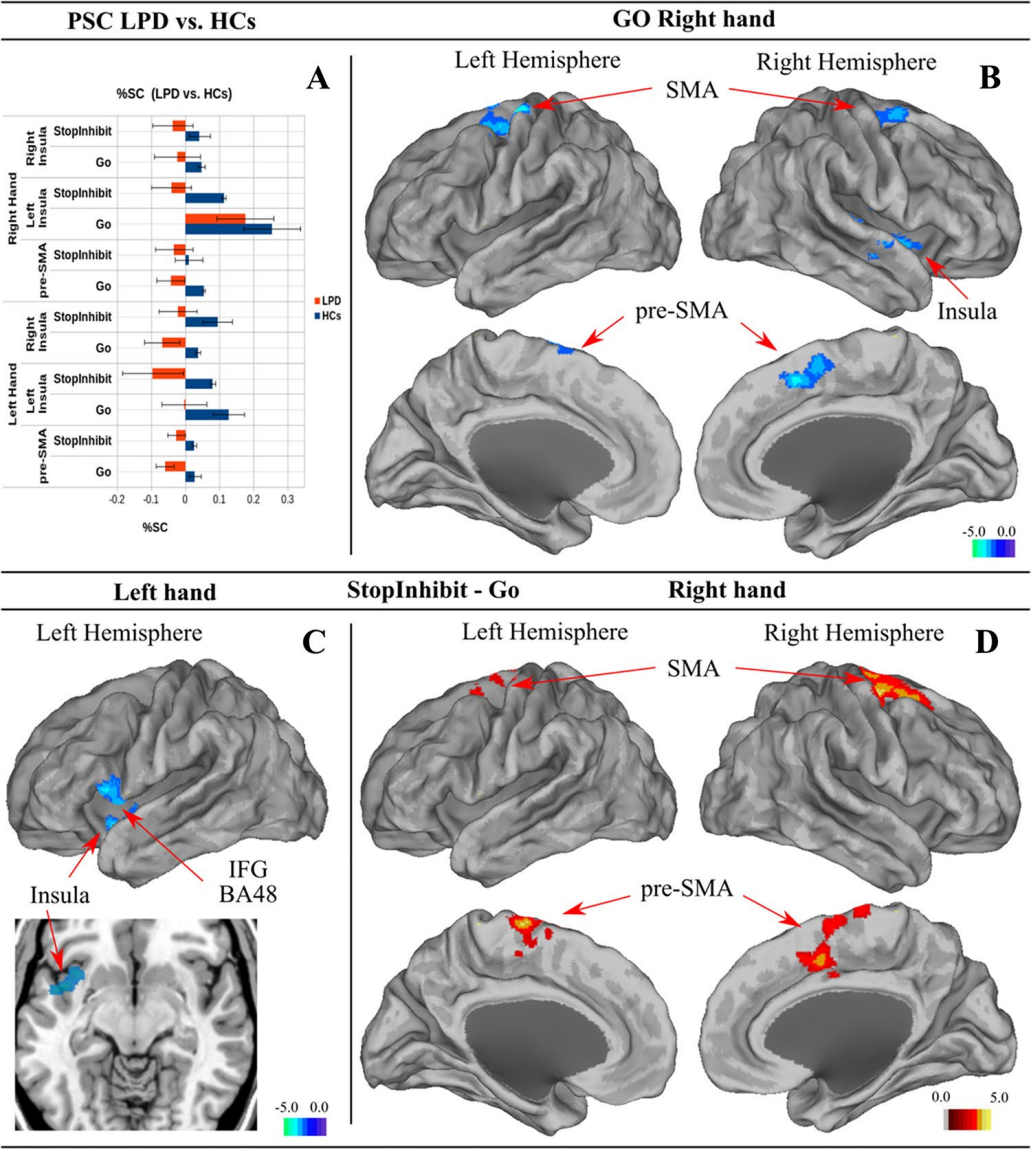
Main effect of Group for the Go and Stop-Inhibit vs. Go contrasts. Hot (red) colors indicate brain regions showing overactivation and cold (blue) colors underactivation in patients with left-sided Parkinson’s disease (PD) compared to healthy controls (HCs). Panel **A**: plot of percent signal change (PSC) for left and right insula and medial pre-SMA for left and right hand. Panel **B**: significant brain regions under-activated in left-sided PD patients compared to HCs during the Go trials for the right hand. Panel C: significant underactivation for Stop-Inhibit vs. Go contrast for the left hand, and panel D: significant overactivation of the patients for the Stop-Inhibit vs. Go contrast for the right hand

For the simple Go events, we did not find significant differences between patients and controls for the most affected left hand. However, for the right hand we found significant differences with BOLD decrements in the right Superior Temporal Gyrus extending to the insula and IFG, the right putamen and pallidum, bilateral Superior Frontal Gyrus (Frontal Eye Fields) and pre-SMA as shown in Fig. 4B and Supplementary Table 4.

#### Stop Inhibit – Go

To further identify the brain areas specifically activated in relation to motor inhibition subtracting the motor component, we examined the Stop-Inhibit-Go contrast controlling for response initiation involved on Go trials. Compared to controls, while using their more affected left hand, LPD patients showed a similar pattern to the Stop-Inhibit contrast, with significant underactivation in the left IFG BA48 (p. Triangularis and Orbitalis) that extends into the insula (Fig. 4C; Supplementary Table 3). Instead, using the less affected right hand, overactivation was found in the SMA (BA6) bilaterally, medial pre-SMA with predominance to the right hemisphere and the right superior frontal gyrus in patients relative to controls as it is shown in Fig. 4D. We described before in the Go contrast, similar brain regions to the Stop-Inhibit vs. Go contrast were underactivated for the right hand. To understand this effect, we measured the Percent Signal Change in the pre-SMA region (shown in Fig. 4A). Thus, in controls the Stop-Inhibit and Go contrast were positive, instead in patients these contrasts were negative. Subtracting a negative value from a negative, we obtained a positive and which we show as overactivation. Therefore, results from this contrast are a consequence of the underactivation of these regions in the LPD group during the Go trials. The Stop-Inhibit vs. Stop-Failure contrast showed a similar pattern than the Stop Inhibit vs. Go contrast (Supplementary Figure S4).

#### SSD (stop-signal delay regressor)

**Fig. 5:**
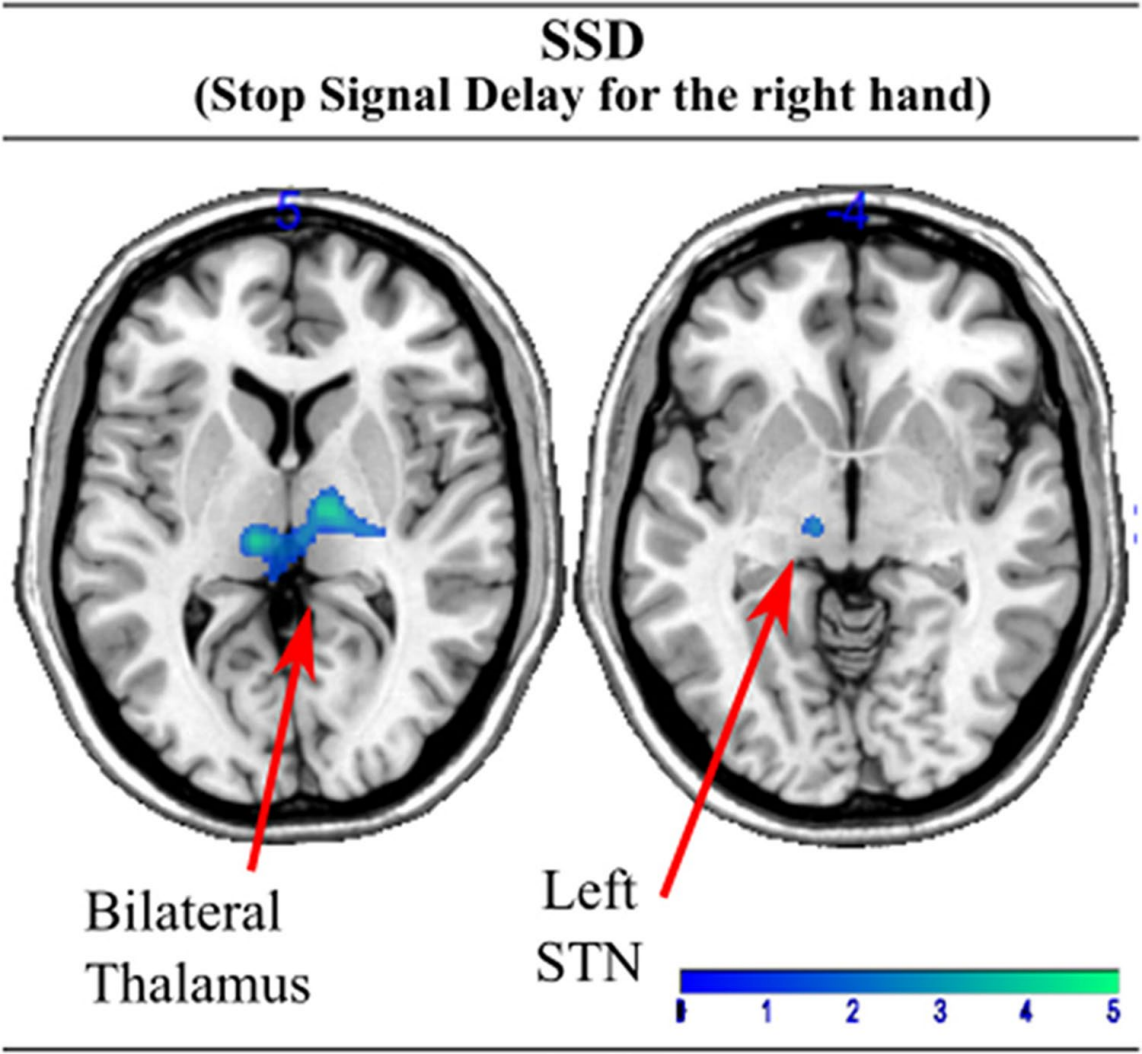
Areas showing underactivation for the left-sided Parkinson Disease group compared with the healthy control group using the SSD (Stop Signal Delay) as regressor. Thus, the successful inhibition trials were weighted with the difficulty level. Figure shows areas of underactivation when patients performed the task with the right hand

This contrast represents brain regions that increase their activity with successful inhibition at higher values of SSD (i.e. the most difficult Stop trials). When performing the task with the right hand, relative to controls, the LPD patients showed underactivation in brain regions including the bilateral thalamus extending to the left STN (Fig. 5, Supplementary table 5). However, we did not find significant differences when performing the task with the left hand.

#### Conjunction analysis between hands

To discern whether LPD alters the most affected hand or both hands during task performance, a conjunction analysis was done in a model including both hands for the main contrasts. Conjunction analysis showed no significant effects for left and right hand together for any of the contrasts of interest. This indicates that abnormal activity it is unique for each condition and hand.

#### PPI

**Fig. 6:**
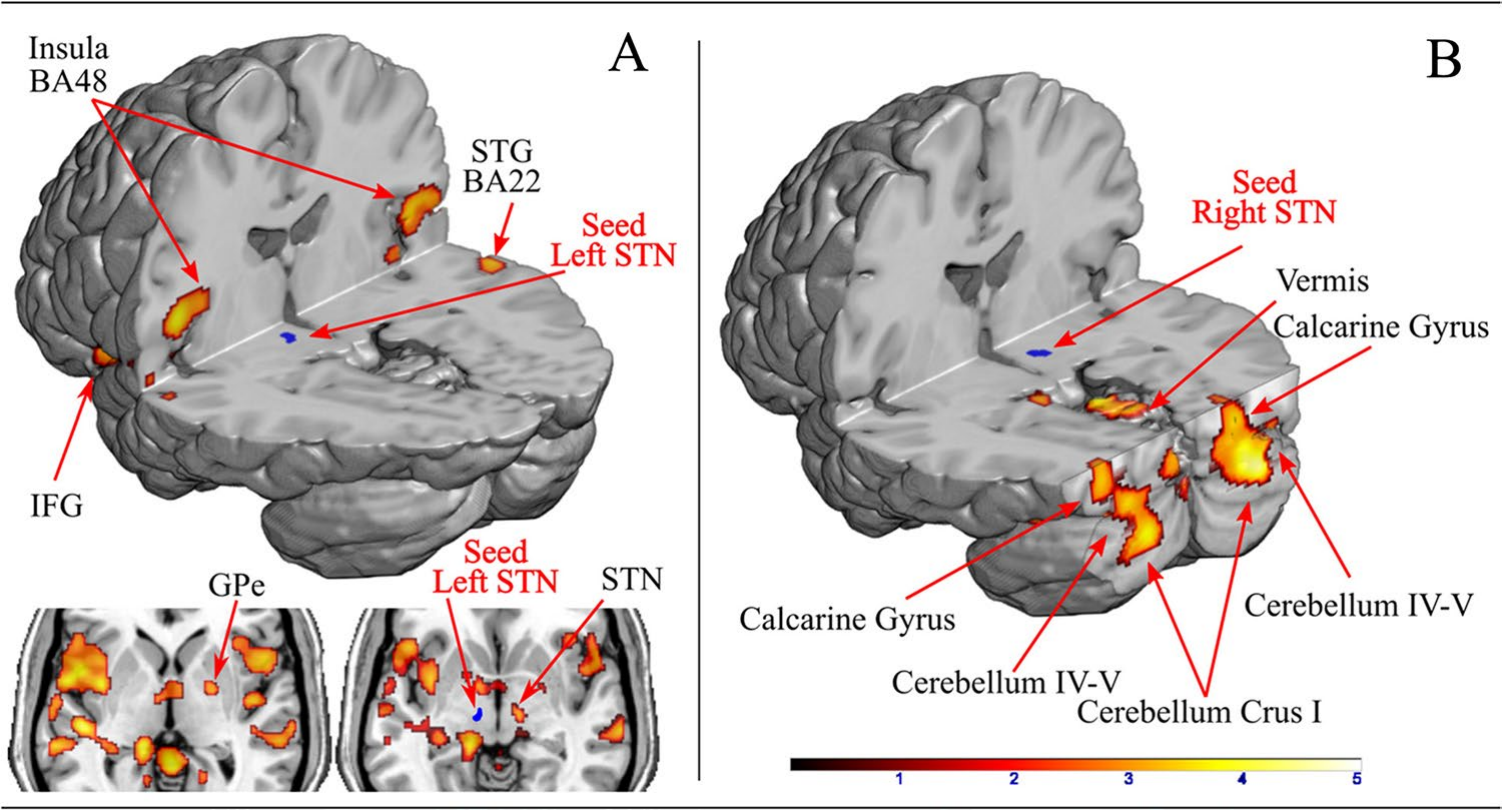
Psychophysiological Interaction differences between patients and controls of the left and right STNs during Stop-Inhibit trials (successful inhibition) with their left hand. Hot colors show significant increases of functional connectivity for the patients. Panel A: connectivity increases of the left STN. Bottom image shows key regions tested using small volume correction (SVC); Panel B: connectivity increases of the right STN. We did not find any significant changes of functional connectivity of the left or right STNs for the right hand

The rationale for performing the PPI analysis with the right and left STN as seeds was to examine the functional connectivity of these key subcortical nodes of the inhibitory network, which are hyperactive in PD [Bergman et al., 1990]. For the Stop-Inhibit vs. Idle contrast, we did not find significant changes in functional connectivity, compared to controls, when patients performed the task with the right hand. In contrast, when performing the task with the more affected left hand we found altered connectivity of both STNs. The ipsilateral left STN seed showed increased functional connectivity with bilateral insula, Rolandic operculum and superior temporal gyrus, left IFG, and right middle temporal gyrus, right lingual gyrus and the cerebellum vermian motor areas (Fig. 6, Supplementary Table 6). Interestingly, we also observed an increased connectivity between the left STN with the right STN and the right globus pallidus (GP) in PD patients on Stop-Inhibit trials. For these anatomically small regions, we used Small Volume Correction (SVC) at peak level P_FWE_ < 0.005, T > 3.10, ROI r = 2 mm), as described in the Fig. 6A bottom image. For the same contrast, when using the left hand, the contralateral right STN in PD patients showed increased functional connectivity with bilateral calcarine gyri and vermian; hemispheric cerebellar cortex comprising sensorimotor lobules IV-V and Crus I, compared to controls (Fig. 6B, Supplementary Table 6) (P_FDR_ < 0.005).

## Discussion

To ascertain the right hemispheric inhibitory network dominance in response inhibition, we examined motor inhibition on a stop-signal task in a selected group of PD patients with predominant LPD motor features. Behaviorally, LPD patients had significantly longer SSRTs compared to age-matched healthy controls, irrespective of whether they were using the right or left hand, indicative of delayed motor inhibition. As impulsivity is one of the prevalent non-motor characteristics in PD (Erga et al., 2017). We found significantly higher scores in PD patients versus healthy controls in line with Aumann et al., 2020). Therefore, to minimize the effects on brain activity of these variables such as drug intake by patients and impulsivity, these variables were incorporated in the fMRI analysis as nuisance factors. Under activation of the left IFG extending to the insula was observed when using the most affected left hand in successful response inhibition compared to controls. When comparing the right vs. left hand, we found patient-specific underactivation of bilateral thalamus extending to the left putamen and pallidum, an underactivation only present when using the most affected left hand for motor inhibition. In LPD, the level of difficulty of inhibitory control (indexed by the SSD) was associated with hypoactivity of the left STN and bilateral thalamus when using the least affected hand (right hand), relative to controls. Finally, PPI analysis revealed significant changes in the functional connectivity of both STNs with cortical (insula, occipital), basal ganglia and hemispheric motor and associative regions of the cerebellum in the PD group relative to controls.

Abnormal response inhibition was seen in LPD compared to controls consistent with prior findings on the stop-signal task [Gauggel et al., 2004; Obeso et al., 2011a]. Similar to our behavioral results, a previous study [Mirabella et al., 2017] also reported on the effect of PD motor symptom laterality on response inhibition and found this to be independent of the more or less affected side. To date, the neural mechanisms that sustain such absent difference remain unknown.

Previous imaging studies have reported the dysfunctional brain circuits mediating altered response inhibition in PD [Manza et al., 2018; Rae et al., 2016; Vriend et al., 2015; Ye et al., 2014a; b]. This evidence, however, has largely overlooked a critical question which is the asymmetric nature of early PD at onset and during many years of evolution, and hence the differential contribution of the right-hemisphere inhibitory network to inhibitory deficits in PD has been uncertain. We show for the first time that successful motor inhibition with the left hand is processed through a network including the left IFG, right putamen and pallidum which is underactive in left-sided PD compared to controls. Consistent with our results, a group of de novo PD patients showed hypoactivation of the left and right IFG compared to controls during successful inhibition on the stop-signal task [Vriend et al., 2015] suggesting that deficient inhibition occurs early even in unmedicated PD but with potential reversal with dopaminergic medication [Manza et al., 2018]. However, medication did not change stop behavior in PD [Obeso et al., 2011b] but in patients who develop dyskinesias, levodopa worsened inhibition associated with the hypo-active right IFG [Cerasa et al., 2015]. The critical importance of the right-hemisphere network in response inhibition is hereby confirmed; and hypoactivity of this network explains deficient stopping behavior as seen in our sample.

Importantly, we provide further evidence on right-hemisphere dominance for motor inhibition on the stop-signal task. PD patients showed hypoactivity over a right-inhibitory network (i.e. IFG and putamen) on difficult Stop trials when using the most affected hand. Moreover, we did not find connectivity changes of either the STN when stopping with the right hand (left hemisphere), compared to controls. Thus, the more affected right-hemisphere in left predominant PD would produce physiological changes in the well-known right-hemisphere inhibitory network.

Differences in the inhibitory while using the more and less affected hands in left-sided PD are suggested by other aspects of the results. The difficulty of motor inhibition as indexed by the SSD regressor for the right hand was associated with under activation in the inhibitory network of the left STN and thalamus relative to controls. In line with the findings of some thalamic nuclei role in adjusting inhibitory control for more difficult timings [Li et al., 2008], this result enhances the view that deficient inhibition in PD is partly explained by the inability to deal with long stop-signal delays which rely on the bilateral thalamus and contralateral STN. These results suggest that at the neural level, delayed motor inhibition in left-predominant PD, shows different patterns of neural adaptation when performing the task with the more or less affected hand.

In healthy participants, successful motor inhibition on the stop-signal or *go no-go* tasks engages a right hemisphere ‘inhibitory network’ including the IFG, ACC and pre-SMA but also the STN, caudate, and thalamus [Aron et al., 2007; Aron and Poldrack, 2006b; Duann et al., 2009; Garavan et al., 1999; Hampshire et al., 2010; Ray Li et al., 2008; Rubia et al., 2003]. In most of these studies, participants used their dominant right hand to perform the experimental task, thus showing ipsilateral right hemisphere activation during action restraint on *go no-go* or motor inhibition on the stop-signal tasks. While patients with predominance of right or left-sided parkinsonism are normally included within the same cohort, it is possible that laterality of motor signs had an effect on motor inhibition. Our study is the first to examine the impact of lateralized parkinsonism on the neural networks engaged by motor inhibition.

The STN as part of the subcortical inhibitory network [Aron et al., 2007; Chen et al., 2020; Mosher et al., 2021] should putatively generate stopping behavior with a leading causal role [Obeso et al., 2014b]. In our PD sample, inhibitory behavior was delayed and slower than controls. Our PPI analysis during successful inhibition with the left hand revealed that for the left STN there was increased functional connectivity with bilateral insular regions. Previous studies have shown the GP to have a role in initiation of actions [Aron and Poldrack, 2006] and to account for early determination of action goals [Arimura et al., 2013], but not in the stopping of actions [Schmidt et al., 2013]. A previous study in PD patients who had had DBS of the GP revealed no direct effect of the modulation of the GP output with “on” versus “off” methodology on motor inhibition on the stop-signal task, but enhanced speed of response initiation [Kohl et al., 2015]. The increased connectivity of the right GP with the left STN during successful motor inhibition with the left hand may reflect the ‘braking’ influence of the STN on the final output pathway of the basal ganglia, the GP. The increased STN-cerebellar connectivity during successful motor inhibition may reflect some form of compensatory activity. Cerebellar activation in PD patients was seen during a *Go no-go* task [Vriend et al., 2015] and patients with focal lesions of the dentate nucleus do exhibit changes in inhibitory paradigms [Olivito et al., 2017]. The cerebellum not only receives input from areas of the cerebral cortex, but also is densely interconnected with the basal ganglia and STN [Bostan et al., 2010; Milardi et al., 2015]. In a study of inhibitory control, a whole-brain age-regression analysis between 10 and 42 years showed a linear age-correlated functional development of right inferior fronto-striato-cerebellar networks during response inhibition and anterior cingulate during error-related processes [Rubia et al., 2007], suggesting that the role of the cerebellum in successful inhibitory control increases with age. Thus, it is possible that the cerebellum also plays a role in motor inhibition in these elderly patients.

There is considerable evidence from a variety of sources for involvement of the cortico-striato-subthalamic-pallidal-thalamic-cortical circuits in reactive, proactive, selective, goal-directed and habitual inhibition [for reviews see Aron, 2011; Jahanshahi et al., 2015]. Using granger-causality analysis of the stop-signal task time-series, a study reported increased connectivity of the pre-SMA with the caudate head and STN during motor inhibition [Duann et al., 2009]. Others have reported increased connectivity between the right IFG and the right caudate in fast inhibitors, while slow inhibitors were characterized by increased connectivity between the pre-SMA and the right caudate [Jahfari et al., 2011]. Recent reports reveal the critical role of ventral STN together with right IFG in allowing or cancelling ongoing actions [Chen et al., 2020; Mosher et al., 2021]. In fact, stopping-related cortical potentials anticipated stopping activity in the STN with synchronized patterns that predicted SSRTs in participants [Chen et al., 2020]. Thus, it seems plausible that the delayed speed of inhibition in PD patients influences the differential engagement of specialized right inhibitory networks and necessitates recruitment of new brain circuits such as motor cerebellar or ventral STN regions for patients to achieve adequate motor inhibition.

## Conclusions

We show, for the first time, the neural correlates of delayed motor inhibition with the right and left hands in patients with left predominant PD. There were important differences in the patterns of brain activation during motor inhibition between hands. For the less affected right hand we found slight under activation of the right inhibitory network, however for the more affected left hand the delayed inhibition was associated with significant under activation of the right inhibitory network and altered functional connectivity of both STNs, requiring engagement of additional regions through strengthening connectivity with cerebellar and other brain structures, as compensatory mechanism for the execution of the task. The results promote further understanding of the neural substrates of delayed motor inhibition in PD.

## Supplementary Information

Below is the link to the electronic supplementary material.

Supplementary file1 (DOCX 3780 KB)

## Data Availability

The data generated or analyzed during this study are included in this published article and its supplementary information files.

## Abbreviations

(LPD): Left-predominant Parkinson disease
(FMRI): Functional magnetic resonance imaging
(SSRT): Stop-signal reaction time
(SSD: Stop-signal delay
