# Supplementary material for: The motor inhibitory network in patients with asymmetrical Parkinson’s disease: An fMRI study
Source: Brain Imaging Behav. 2022 Jan 12;16(3):1349–61. doi: 10.1007/s11682-021-00587-5 (PMC9107438; doi:10.1007/s11682-021-00587-5)
Supplement: Supplementary file 1 — Supplementary file1 (DOCX 3780 KB) [file 11682_2021_587_MOESM1_ESM.docx]

**The motor inhibitory network in patients with asymmetrical Parkinson’s disease:**

**An fMRI study.**

***Supplementary material***

***fMRI acquisition and analysis***

Imaging was performed using a 3-Tesla scanner (Trio-TIM, Siemens AG, Erlangen, Germany) equipped with a 12-channel head coil array. Visual stimuli were projected onto a screen behind their head. Stimulus presentation and response recording were controlled using Cogent (Cogent 2000, Wellcome Department of Imaging Neuroscience, UCL, London, UK) and Matlab v7.9 (Mathworks Inc., Natick, USA). Eye movements were monitored and registered using an eye-tracking device (ASL, Bedford, MA). A T2*-weighted (Echo Planar Imaging - EPI) sequence sensitive to BOLD contrast was used to acquire ~300 volumes of the experiment. Each volume comprised 45 transverse slices with a 15% gap, covering the entire brain. Other imaging parameters were: resolution=3mm isotropic, echo time=30ms and repetition time=3.0s. We also acquired a high resolution anatomical image with an MPRAGE sequence.

Data were analysed using “Statistical Parametric Mapping” software, version SPM8 (Wellcome Department of Imaging Neuroscience, UCL, London, UK). For preprocessing purposes, we used Diffeomorphic Anatomical Registration through Exponentiated Lie algebra toolbox (DARTEL) (Ashburner 2007). The DARTEL toolbox provided a high-dimensional normalization protocol expected to increase registration accuracy, thereby increasing sensitivity and improving localization in our comparisons.

In the first preprocessing step, anatomical images for all subjects were segmented into gray matter (GM), white matter (WM), and cerebrospinal fluid using the unified segmentation tool (Ashburner and Friston 2005) provided in SPM8. The GM and WM segments were input into DARTEL toolbox in order to create a customized template for all participants in the study. DARTEL then registered the individual tissue segments to the template in order to obtain the individual nonlinear deformation fields. After inter-subject registration, all the GM and WM maps were further transformed into the Montreal Neurological Institute (MNI) space, modulated to compensate for spatial normalization effects.

For the second step, the ~300 volumes of the experiment were realigned to the first volume, corrected for bias field inhomogeneities and co-registered to the anatomical image. Subsequently, fMRI volumes were brain extracted using custom scripts in Matlab. Next, the individual tissue deformations obtained were then used to warp and modulate each participant’s fMRI data for nonlinear effects. Finally, the modulated fMRI volumes were smoothed with an 8 mm^3^ FWHM Gaussian kernel and filtered over time using a high-pass filter of 128s.

Subsequent statistical analyses were performed using a general linear model to estimate the effects at each voxel of the brain (Friston et al. 1995). Additionally to the tasks, we constructed an extra regressor with the corresponding SSD value for each Stop-Inhibit event. The SSD regressor weights the events according to the difficulty level, where successful inhibition with higher SSD values were considered more difficult to inhibit than those with lower SSD values. Omission errors were classified as errors for the fMRI analysis. Thus at the first level, the time series of each participant was modelled with the following tasks as an event related design: Go, Stop-Inhibit, SSD regressor, Stop-Respond, Idle and Errors, convolving each event with a canonical double gamma as a hemodynamic response function (HRF). Onset times for each event corresponded to the presentation of the visual cue (arrow), not discriminating between key presses. At this level, we estimated the following contrasts of interest for each participant and hand: Go vs. Idle and Stop-Inhibit vs. Idle, Stop-Inhibit vs. Go, and SSD regressor.

At the second level, for each contrast of interest we used two-way ANOVA with Group (patients and healthy controls) and Hand as factors. We tested for each contrast between group differences in BOLD signal.

Additionally, we performed a Psychophysiological Interaction analysis (PPI) with the objective to assess variations in effective connectivity of the STN as the ‘seed’ or index area during the Stop-Inhibit vs. Idle events (psychological factor). PPI is able to detect a significant change in the contribution of one index region to another taking into account the empirical or psychological context (Friston et al., 1997). Using this method, we were able to identify brain regions which showed enhanced or weakened functional connectivity with the STN index areas (left and right). For the PPI analysis, we used the procedure described in Stephan (Stephan, et al. 2003), selecting two seeds using two STN masks: one from the left and one from the right STN. For each seed, we extracted the individual temporal series for the left hand. Subsequently, time series *y_o_* were mean corrected and high pass filtered. The PPI regressor was estimated as the element-by-element product of the extracted time series *y_o_* and a vector *u* coding for the main effect of the task (Stop-Inhibit vs. Idle) for the left hand. The PPI regressor (*y_o_×u*), the psychological variable *u*, the physiological variable *y_o_* and the constant term were included as regressors in a first level analysis. Individual t-contrasts were computed as 1 for the PPI regressor and 0 elsewhere. Contrasts obtained in the individual PPI analyses for each STN corresponding only to the left hand were introduced in a two-way ANOVA with factors group (Hcs and LPD) and STN (ipsilateral and contralateral to the left hand), to compare the connectivity maps between the two groups of participants and STNs' effects. In all cases, BOLD and PPI group analyses used the p<0.01 FDR cluster corrected (Bennett et al 2009).

**Results**

**Behaviour**

To test the assumption of the race horse model about the independence of the stop and go processes, additional analyses were performed in each group. To test whether independence between go and stop processes was present, direct comparisons between Go RT and Stop-Respond RT were made within groups and between hands. As expected, based on the distribution probabilities of both trial types (Figure S1), faster Stop-Respond RTs compared to Go RTs were seen in LPD for the left [*t*_(13)_=5.46, *p*<0.001] and right hands [*t*_(13)_=4.86, *p*<0.001]. Controls revealed a similar effect for left [*t*_(25)_=8.92, *p*<0.001] and right hands [*t*_(25)_=8.92, *p*<0.001]. However, correlations between initiation (Go trials) and inhibition of actions (SSRT) were positive and significant in some task performance including LPD with their left [*r=*.93, *p*<0.001] and right hands [*r=*.82, *p*=0.001]. Meanwhile, controls showed only a significant correlation while using their right hand [*r=*.46, *p*=0.01] but not with the left hand [*r=*.07, *p*=0.70]. Based on the failure to confirm full independence of going and stopping in our data, we interpret the deviation from 50% inhibition, a possible reason for observing links between going and stopping. Yet, the results confirm one of the expectations of the model (Go RTs > Stop-Respond RTs) and we selected to use the integration method for estimation of response inhibition which reduces the SSRT overestimation when violations of the model occur (Verbruggen et al., 2019).

**Go vs. Idle trials**

Relative to healthy controls, on the Go trials, significant main effects were found. Post-hoc comparisons showed differences between patients and controls in BOLD data only for the right hand. Relative to the controls, the LPD patients showed a significant underactivation in the. pre-SMA (BA6), the right insula, among other areas (Figure S2; Supplementary Table 1) with the less affected hand. No significant areas of overactivation were identified with the threshold used*.* We did not find any significant differences in brain activation between patients and healthy controls for this contrast with the left hand.


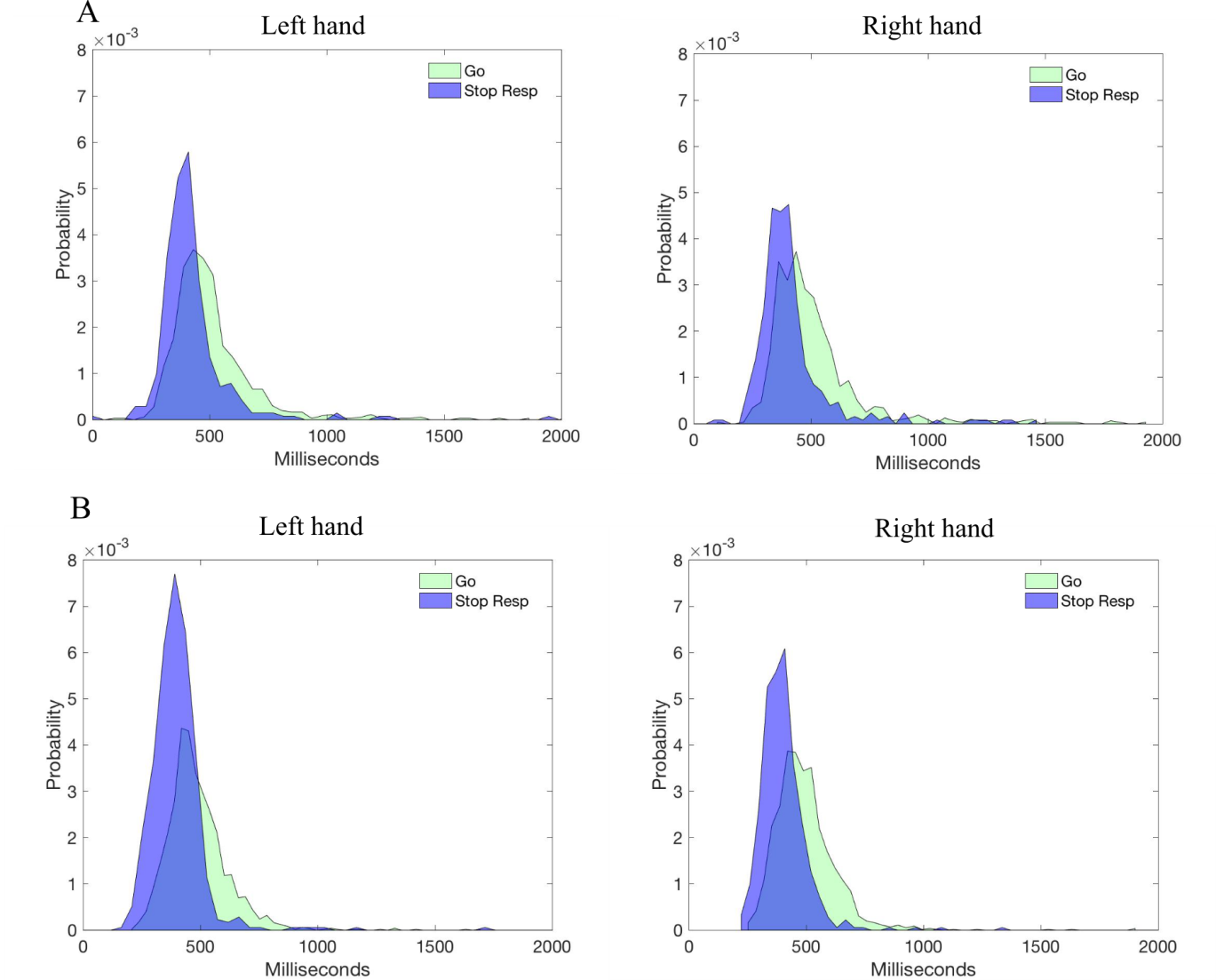


***Figure S1.*** *Distribution probabilities for Go and Stop-Respond trials. A, Distribution curves for LPD group for left and right hand respectively; B, Distribution curves for the control group for left and right hand respectively.*

*
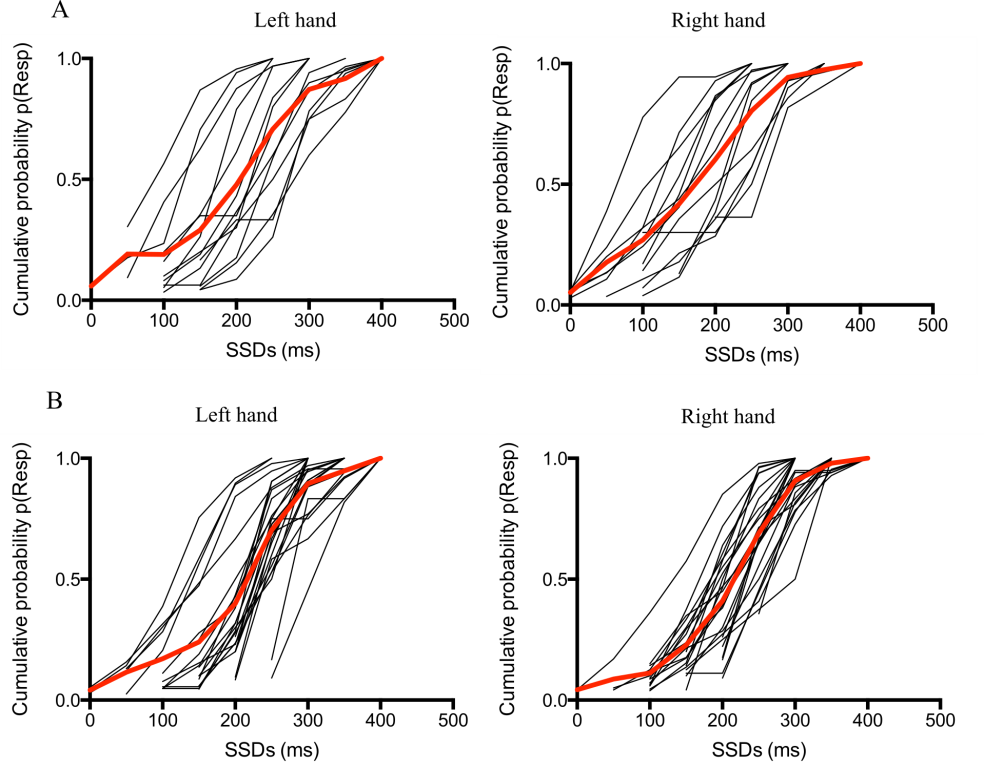
*

***Figure S2.*** *The cumulative probability of response inhibition failure as SSD value increases. The basic assumption of the race model expects as the SSD increases, stop failure rate to increase. Cumulative probability of responding curves for (A) LPD and (B) controls for left and right hand respectively. Each graph represents individual inhibition probabilities with average group values marked in red.*

*Neuroimaging*


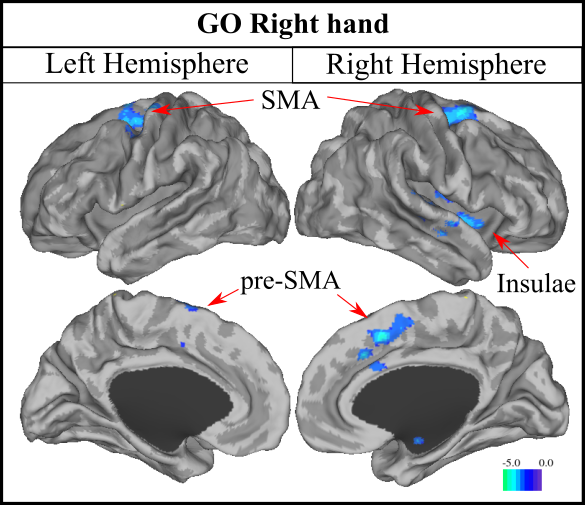


***Figure S3.*** *Brain regions showing significant underactivation in patients with left sided Parkinson’s disease relative to healthy controls (T1 template) during the Go trials when performing the task with the right hand. Contrast evaluated at p>0.01 FDR cluster correction. No significant areas of overactivation were identified with this threshold.*

***
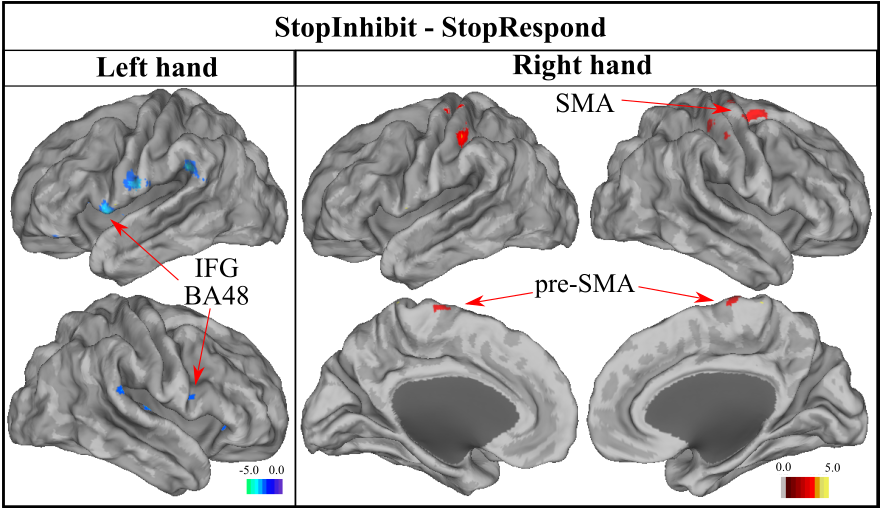
***

***Figure S4.*** *Figure shows brain regions overactivated for the right hand in hot colors, and underactivated for the left hand in cold colors, in patients with left sided Parkinson's disease compared to controls for the contrast Stop-Inhibit vs. Stop-Respond. The contrast was evaluated for p<0.005 uncorrected.*

***
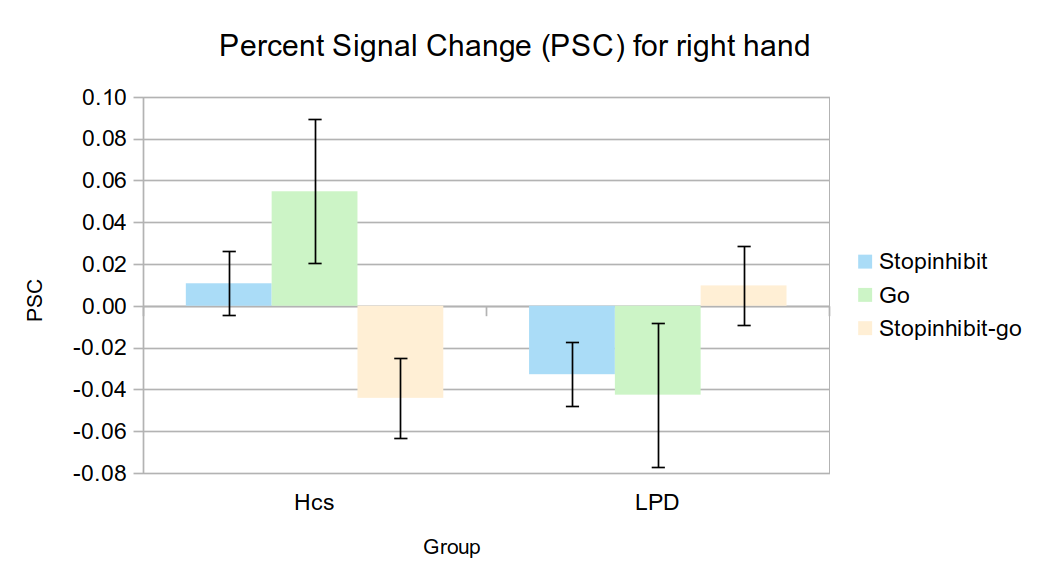
Figure S5.*** *The average of the Percent Signal Change of the right medial SMA when performing with the right hand for the evaluation of the three contrasts: Stop-Inhibit, Go and Stop-Inhibit vs. Go; Hcs=healthy controls, LPD=PD with left-dominant disease*

***Barratt Impulsiveness Scale, BIS-11***

The BIS-11 is a self-administered scale consisting of 30 questions related to manifestations of impulsivity and built from the theoretical model of Ernst Barratt. In the present work, we applied the version validated for Spain by Oquendo et al (2001). The instrument corresponds to a Likert scale with four possible answers: never or almost never (0), occasionally (1), Often (3) and almost always / always (4). The score of this scale ranges from 30 to 120 points, higher scores indicating the presence of impulsive behaviors. The instrument provides scores on three subdomains of impulsivity: cognitive impulsivity interpreted as a tendency to make quick decisions after little consideration; motor impulsivity that refers to the propensity of the person to react to the stimulus of the moment without considering the consequences; and finally non-planned impulsivity defined as the tendency to fail to plan future action and increased interest in the present situation than the future. The Total Impulsivity corresponds to the arithmetic sum of all subdomains described before.

***
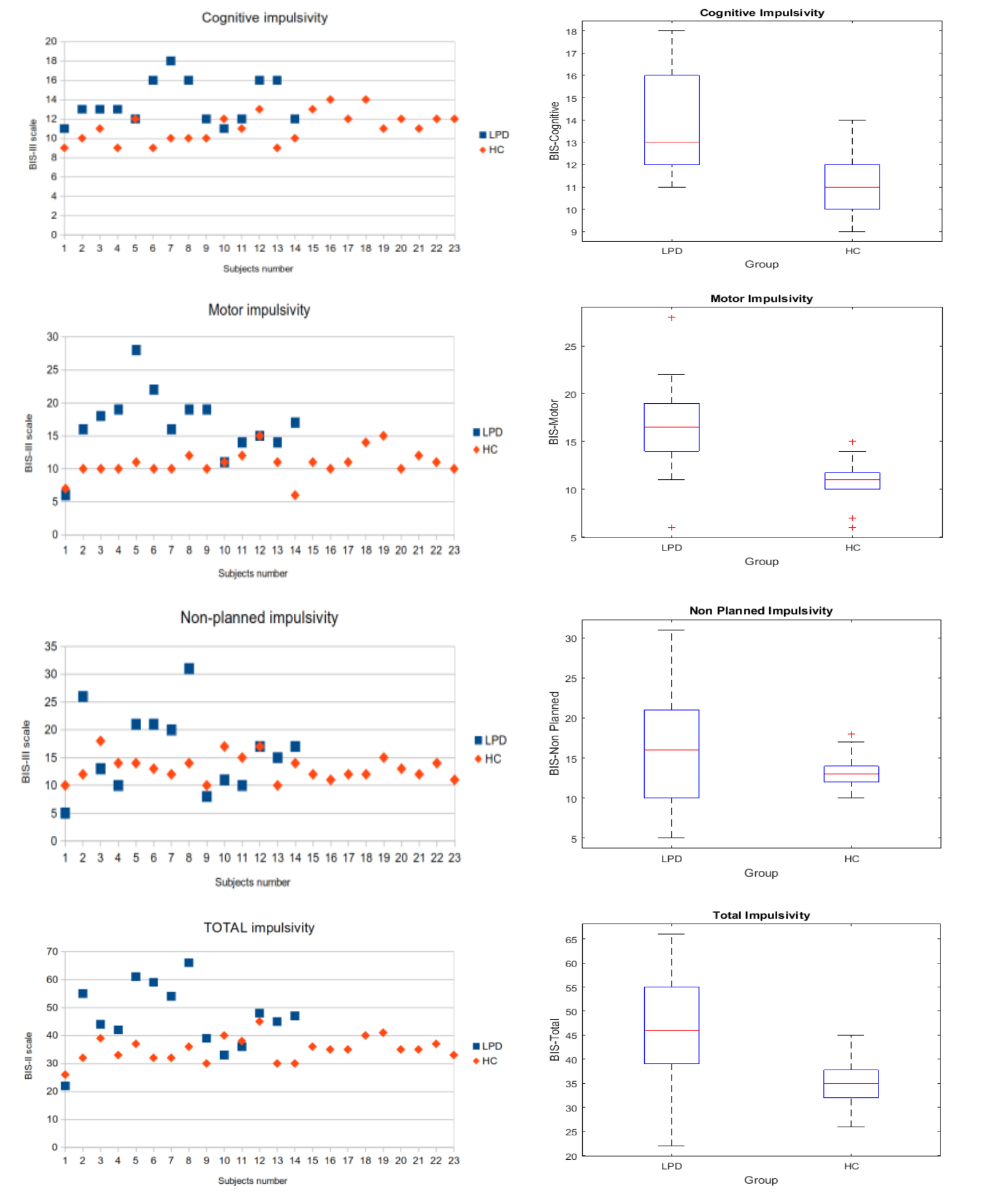
***

***Figure S6***. Left panel: s*catter plot of the individual values measured with the BIS-11 instrument; and in the right panel the boxplot of mixed ANOVA between groups.*

***Supplementary references***

Ashburner J. 2007. A fast diffeomorphic image registration algorithm. Neuroimage. 38(1):95-113.

Friston KJ, Holmes a. P, Worsley KJ, Poline J-PP, Frith CD, Frackowiak RSJ. 1995. Statistical Parametric Maps in Functional Imaging: A General Linear Approach. Hum Brain Mapp. 2:189–210.

Barrat ES. Impulsiveness and agression. Violence and mental disorder Development in risk assessment En: Monahan J. Steadman HJ. EDS. Chicago: The University of Chicago Press. 1995; 61-79.

Oquendo MA, Baca-Garcia E, Graver R, Morales M, Montalban V, Mann JJ. Spanish adaptation of Barrat Impulsiveness Scale (BIS). Eur J Psychiatry 2001; 185:147-155.

***Supplementary Table 1.*** *Areas showing significantly lower activation in patients with left–sided Parkinson’s disease compared to healthy controls during Stop-Inhibit trials, when using the left hand.*

| **Region** | **MNI Coordinates** | | | **t value** | **Cluster Size** |
| --- | --- | --- | --- | --- | --- |
|  | X | Y | Z |  |  |
| Left Anterior Insula | -29 | 20 | -9 | 3.35 | 2660 |
| Left IFG (p. Triangularis) BA44 | -50 | 15 | 9 | 4.53 |  |
| Left IFG (p. Orbitalis) | -35 | 14 | 9 | 3.72 |  |
| Left IFG (p. Orbitalis) | -44 | 45 | -6 | 3.72 |  |
| Left Temporal Pole | -53 | 9 | 2 | 4.86 |  |

***Supplementary Table 2.*** *Brain regions underactivated during successful inhibition for the more affected left hand. After post-hoc comparisons we also tested for hand differences between groups. Thus, for healthy controls we did not find significant hand differences in brain activity during successful inhibition, instead for the left-sided Parkinson patients we found significant underactivation for the in the left hand.*

| **Region** | **MNI Coordinates** | | | t value | **Cluster Size (voxels)** |
| --- | --- | --- | --- | --- | --- |
|  | X | Y | Z |  |  |
| Left Thalamus prefrontal | -9 | -27 | 6 | 4.58 | 3303 |
| Left Medial Cingulate Cortex | -14 | -32 | 53 | 4.28 |  |
| Left Thalamus prefrontal extended to left Putamen and internal capsule | -14 | -18 | 6 | 4.34 |  |
| Right Thalamus prefrontal | 11 | -24 | 6 | 4.1 | 1276 |
| Right Thalamus temporal | 12 | -36 | 8 | 3.37 |  |
| Right Putamen | 23 | 6 | 14 | 3.28 |  |
| Right Pallidum | 18 | -5 | 3 | 3.23 |  |

***Supplementary Table 3.*** *Areas showing significant decreased or increased activation in patients with left-sided Parkinson’s disease relative to healthy controls for the Stop-Inhibit vs. Go contrast, when using the left or right hands.*

| Left Hand | | | | | | Right Hand | | | | | | | |
| --- | --- | --- | --- | --- | --- | --- | --- | --- | --- | --- | --- | --- | --- |
| **Decrease activity of PD compared to HCs for Stop-Inhibit vs. Go task for left hand (Cluster size 1429 vox)** | | | | | **Increase activity of PD compared to HCs for Stop-Inhibit vs. Go task for right hand (Cluster size 4538 vox)** | | | | | | | | |
| Region | t-value | X | Y | Z |  | | Region |  | t-value | X | Y | Z |  |
| L IFG (p. Triangularis) | 4.27 | -50 | 15 | 9 | R Superior Frontal Gyrus | | | | 5.21 | 27 | -8 | 59 |  |
| L IFG (p. Orbitalis) | 3.86 | -33 | 14 | -17 | L Posterior-Medial Frontal (SMA) | | | | 4.06 | -5 | -14 | 71 |  |
| L Temporal pole | 3.63 | -50 | 5 | 2 | R Posterior-Medial Frontal (SMA) | | | | 3.69 | 3 | -2 | 66 |  |
| L Insula Lobe | 3.34 | -44 | 3 | -5 | R Superior Frontal Gyrus FEF | | | | 3.89 | 20 | 6 | 60 |  |
|  | | | | | L Precentral Gyrus | | | | 3.56 | -32 | -9 | 66 |  |
|  |  |  |  |  | L Superior Frontal Gyrus FEF | | | | 3.48 | -20 | 6 | 59 |  |

***Supplementary Table 4.*** *Areas showing significantly underactivation in patients with left–sided Parkinson’s disease compared to healthy controls during Go trials, when using the right hand.*

| **Region** | **MNI Coordinates** | | | t value | **Cluster Size (voxels)** |
| --- | --- | --- | --- | --- | --- |
|  | X | Y | Z |  |  |
| Right Superior Temporal Gyrus | 48 | -12 | -9 | 4.52 | 1802 |
| Right putamen | 32 | -11 | 9 | 3.64 |  |
| Right pallidum | 23 | -3 | 11 | 3.41 |  |
| Right IFG (p. Orbitalis) | 41 | 17 | -11 | 3.26 |  |
| Right Superior Frontal Gyrus (FEF) | 14 | 6 | 56 | 5.06 | 1502 |
| Right Superior Medial Gyrus (SMA) | 9 | 18 | 48 | 4.21 |  |
| Right Precentral Gyrus | 26 | -12 | 60 | 3.47 |  |
| Left Precentral Gyrus | -33 | -14 | 66 | 4.08 | 1380 |
| Left Superior Frontal Gyrus (FEF) | -20 | -6 | 57 | 3.83 |  |
| Left Middle Frontal Gyrus (SMA) | -20 | 3 | 56 | 3.54 |  |

***Supplementary Table 5.*** *Areas showing significant decreased activation in patients with left-sided Parkinson’s disease relative to healthy controls for the more difficult trials during the successful stop signal task (Stop Signal Delay contrast regressor), when subjects performed the task with the left or right hands.*

| Right Hand | | | | | | | |
| --- | --- | --- | --- | --- | --- | --- | --- |
| **Decreased activity of PD compared to Hcs for the SSD regressor with the right hand (Cluster size 1280 vox)** | | | | | | | |
|  | Region |  | t-value | X | Y | Z |  |
| Right Thalamus Prefrontal | | | 4.55 | 12 | -12 | 5 |  |
| Left Thalamus Prefrontal | | | 4.5 | -11 | -23 | 3 |  |
| Right Thalamus Prefrontal | | | 3.77 | 5 | -21 | 6 |  |
| Left STN | | | 3.33 | -8 | -18 | -9 |  |

***Supplementary Table 6.*** *The results of psychophysiological interactions with the left and right subthalamic nucleus (STN) used as seeds. Left panel: areas showing increased connectivity with the left STN for PD patients compared to HCs for the Stop-inhibit-Idle contrast, when using the left hand. Right panel: areas showing increased connectivity with the right STN for PD patients compared to HCs for the Stop-Inhibit vs. Idle contrast, when using the left hand.*

| Left STN | | | | | | Right STN | | | | | | | |
| --- | --- | --- | --- | --- | --- | --- | --- | --- | --- | --- | --- | --- | --- |
|  | t-value | X | Y | Z |  | |  |  | t-value | X | Y | Z |  |
| L Rolandic Operculum | 4.69 | -48 | 3 | 6 | L Calcarine Gyrus | | | | 4.78 | 2 | -96 | 0 |  |
| L Superior Temporal Gyrus | 4.34 | -47 | -22 | 1 | R Calcarine Gyrus | | | | 3.90 | 5 | -84 | 9 |  |
| L Insula Lobe | 3.97 | -38 | 2 | -2 | R Cerebellum (Crus 1) | | | | 4.55 | 29 | -80 | 26 |  |
| L IFG (p. Orbitalis) | 3.37 | -48 | 21 | -9 | L Cerebellum (IV-V), | | | | 4.03 | -26 | -46 | -21 |  |
| Cerebellar Vermis (4/5) | 4.00 | 2 | -43 | 0 | L Cerebellum (Crus 1) | | | | 4.03 | -33 | -76 | -29 |  |
| R Lingual Gyrus | 3.52 | 12 | -65 | 8 | L Cerebellum (VI) | | | | 3.86 | -41 | -58 | -23 |  |
| R Rolandic Operculum | 4.52 | 59 | -10 | 15 | R Cerebellum (VI) | | | | 3.74 | 23 | -69 | -18 |  |
| R Superior Temporal Gyrus | 3.84 | 50 | -25 | 4 | Cerebellar Vermis (IV-V) | | | | 5.40 | 0 | -46 | 1 |  |
| R Middle Temporal Gyrus | 3.55 | 68 | -22 | -9 | L Precuneus | | | | 4.40 | -11 | -39 | 3 |  |
| R Insula Lobe | 3.40 | 45 | -7 | 0 | L Lingual Gyrus | | | | 3.02 | -11 | -32 | -6 |  |
